# Streamlined stable CHO cell line development using droplet microfluidics with image-based monoclonality assessment

**DOI:** 10.1007/s00449-026-03406-7

**Published:** 2026-08-19

**Authors:** Kumar Vishven Naveen, Akanksha Tyagi, Omnia Mohammed Hamid Ibrahium, Rainer E. A. W. Fischer, Raluca Ostafe

**Affiliations:** 1 Purdue Institute of Inflammation, Immunology and Infectious Disease, Purdue University, West Lafayette, 47907 USA; 2 Department of Biological Sciences, Purdue University, West Lafayette, IN 47907 USA

**Keywords:** FRET secretion assay, Picodroplet screening, Antibody secretion, ExpiCHO-S, High-throughput clone screening

## Abstract

Rapid generation of monoclonal CHO cell lines remains a key bottleneck in mammalian cell line development (CLD), often requiring months of iterative screening and cloning. Here, we present an integrated droplet microfluidic workflow for high-throughput screening and isolation of antibody-producing ExpiCHO-S cells using a FRET-based secretion assay. The FRET assay was developed and optimized for picodroplet-based screening by evaluating probe configuration, donor-to-acceptor ratio, antibody concentration range, and signal saturation behavior. The platform enables picodroplet-based detection of secreted antibodies, sequential enrichment of antibody-producing cells, and image-verified single-cell isolation. Application of this approach yielded image-verified monoclonal antibody-producing clones that maintained consistent antibody production during the stability assessment performed in this study, including in the absence of selection pressure. By reducing the early clone-identification phase from DNA transfection to validated 96-well antibody-producing clones to approximately 5 weeks, this workflow addresses key limitations of conventional approaches and provides both a practical FRET assay-development framework and a scalable strategy for streamlining early CLD workflows in biomanufacturing.

**Supplementary Information:**

The online version contains supplementary material available at https://doi.org/10.1007/s00449-026-03406-7.

## Introduction

Stable mammalian cell line development (CLD) is a critical upstream step in biomanufacturing and underpins the production of recombinant proteins, monoclonal antibodies, and other biologics. Chinese hamster ovary (CHO) cells remain the dominant host for industrial production due to their adaptability, scalability, and ability to support complex protein expression. Within this workflow, rapid identification of productive monoclonal cell lines remains a major bottleneck, as cell line quality, clonality, and production stability directly influence downstream process development and manufacturing efficiency.

**Fig. 1:**
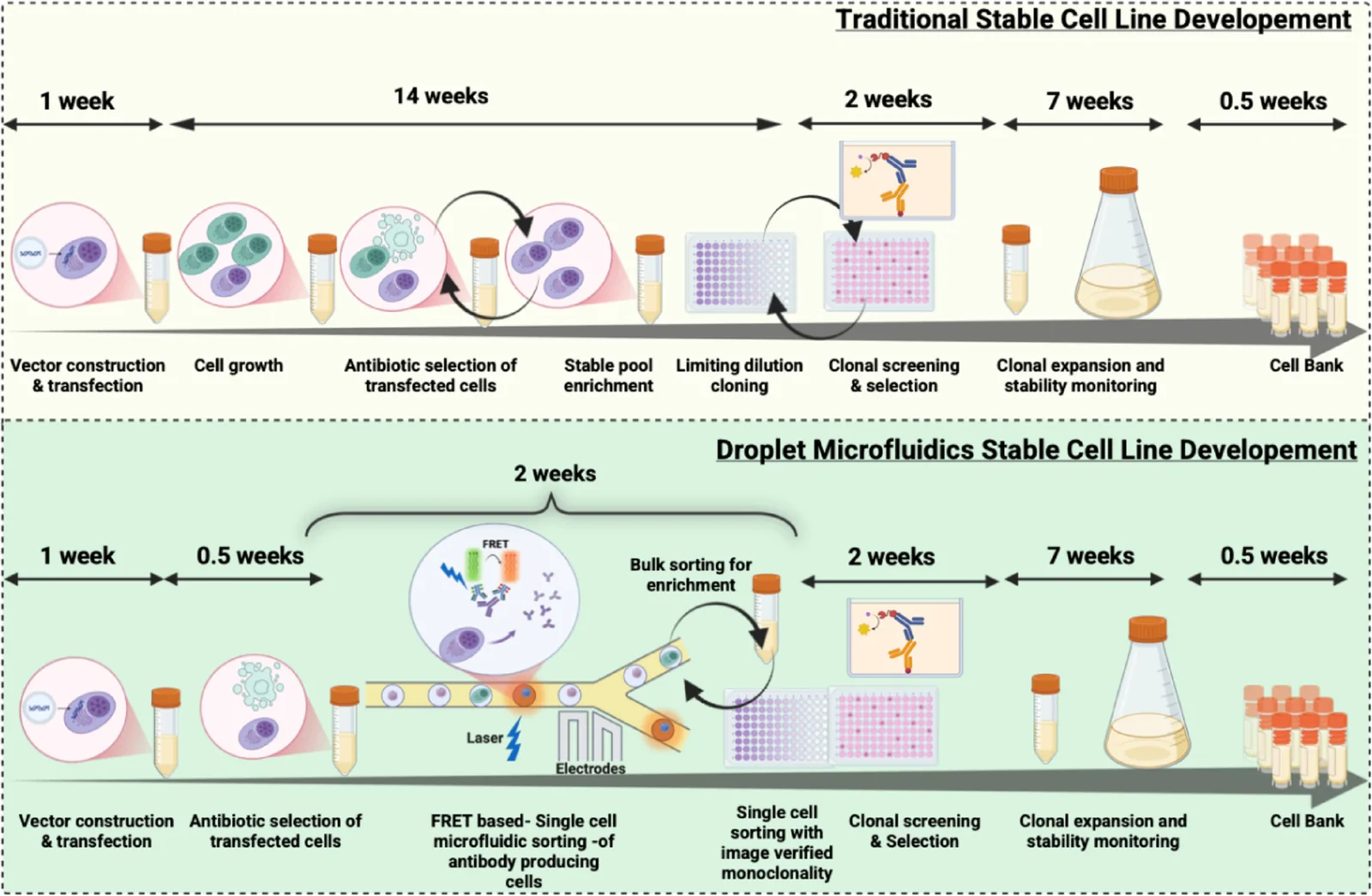
Schematic comparison of the traditional stable cell line development workflow (upper panel) with the droplet microfluidics-assisted workflow (lower panel), highlighting increased throughput and a shorter early clone-identification timeline from DNA to validated 96-well antibody-producing clones. Downstream clonal expansion, cell line stability monitoring, cell banking, and process-development steps were not optimized in this study and are not expected to differ substantially after clone identification between conventional and droplet microfluidics-assisted CLD workflows. Image created via BioRender.com

Traditional workflows-encompassing limiting‑dilution cloning (LDC), rely on Poisson statistics for single-cell deposition; however, a single round does not guarantee monoclonality; consequently, multiple rounds of LDC, prolonged clonal outgrowth, repeated productivity screening, and cell line stability assessment are typically required, significantly extending timelines and increasing resource consumptions [1]. In general, LDC-based approaches spanning transfection, antibiotic selection, semi-solid media screening, and mini‑pool productivity assessment can require 6–12 months to generate a validated, monoclonal, antigen-specific clone suitable for scale-up [2], as described in Fig. 1. Although, modern single‑cell isolation tools like fluorescence‑activated cell sorting (FACS) or image‑based printers (e.g., Cytena) improve single-cell clonality assessment[3, 4], these tools typically do not directly assess secretion level during sorting because secreted antibodies accumulate in the surrounding culture supernatant rather than remaining associated with the producing cell. Consequently, after single-cell deposition, researchers must still expand clones and perform secondary screening to find high producers, adding weeks to the process.

To overcome the inability of conventional single-cell isolation methods to directly link clonality with secretion phenotype, automated opto-fluidic platforms have been developed that enable secretion-based selection at the single-cell level. These systems compartmentalize individual cells into nanoliter-to picoliter-scale micro-wells, allowing secreted antibodies to accumulate in close proximity to the producing cell and enabling direct measurement of secretion during isolation [5].

These systems represent a substantial improvement over conventional FACS- or limiting dilution-based workflows by combining clonality assurance with secretion-linked selection. However, practical limitations in throughput, assay flexibility, and accessibility can restrict broader implementation, particularly in workflows requiring efficient screening of heterogeneous early transfection pools [6].

These limitations have driven growing interest in droplet-based microfluidic approaches, which maintain secretion-linked single-cell compartmentalization while offering substantially higher throughput and flexible assay modalities and are therefore particularly well-suited to the statistical problem of finding rare, high-secreting cells within heterogeneous early transfection pools [7, 8].

Droplet microfluidics has been broadly applied to enzyme-directed evolution experiments [9–11] and has increasingly been used for antibody discovery, including antigen-specific B-cell isolation, antibody-secreting cell screening, antibody sequencing, and target-cell binding assays [12–18]. These studies demonstrate the power of droplet microfluidics for high-throughput protein discovery and functional screening. However, most antibody-discovery workflows are designed to identify antigen-specific antibodies, requiring specialized assay formats for each antigen or protein target of interest. In addition, these workflows generally focus on discovery rather than generation of stable recombinant CHO cell lines, and the assay-development process is frequently not described in sufficient detail to guide transfer to other protein targets or stable CHO CLD applications.

To address these gaps, integrated platforms have emerged that combine droplet generation, incubation, secretion detection, and single-cell recovery within a automated workflow [19, 20]. In this study, we employ the Cyto-Mine^®^ platform as one such implementation, encapsulating individual cells into ~ 450-pL droplets to allow accumulation of secreted antibody. After incubation, secretion is quantified using a fluorescence resonance energy transfer (FRET)-based total IgG assay, enabling sorting of droplets containing antibody-producing cells. During deposition into microtiter plates, each droplet is imaged to support image-based single-cell verification, allowing recovery of verified single cells for downstream clonal expansion.

Implementation of secretion-based FRET assays in picoliter droplets presents additional assay-development challenges, including probe-to-analyte stoichiometry, signal saturation, and reduced dynamic range under confined secretion conditions. Rapid accumulation of secreted antibody within the confined droplet volume can shift the probe-to-analyte stoichiometry during incubation, resulting in signal saturation, probe competition, or hook-like effects. Under such conditions, high-producing cells may generate FRET signals overlapping with those of low- or non-producing cells, potentially leading to incorrect ranking and loss of productive clones during screening. Despite the increasing use of droplet-based secretion assays, the effects of probe format and donor-acceptor stoichiometry on the dynamic range required for single-cell secretion screening remain insufficiently described. Thus, beyond the cell-sorting workflow itself, practical assay-development guidance is needed to enable reliable transfer of FRET-based droplet screening to new antibody formats, secretion levels, and CLD settings. The current work addresses this gap by defining assay-development parameters required for FRET-based picodroplet screening.

In addition to assay optimization, the timing of secretion-based selection is important for early CLD workflows. In conventional approaches, functional productivity screening is often performed only after antibiotic-selected pools or clones have recovered sufficiently for expansion. In contrast, the workflow evaluated here applies FRET-based enrichment early during antibiotic selection, thereby combining selection for Zeocin resistance with functional enrichment for antibody secretion. Because early post-transfection populations may still contain cells with transient expression or reduced viability, FRET-positive cells from the first sort were collected in bulk for enrichment and further recovery rather than treated as final clone outputs. Continued Zeocin selection, recovery/expansion, a second FRET-based single-cell deposition step, ELISA screening, and subsequent stability assessment were then used to identify stable antibody-producing clone candidates.

By integrating secretion-based screening with image-verified single-cell recovery, this approach enables early identification of antibody-producing clones at the 96-well stage in approximately 5 weeks (~ 35–37 days), as summarized in. Subsequent clonal expansion, cell line stability monitoring, and cell banking remain downstream workflow steps.

Stable CLD may employ a range of expression vector and genomic integration strategies, including lentiviral, transposon-based, site-specific recombination, and gene amplification approaches [21–25]. In practice, expression vector designs and integration strategies are frequently proprietary and implemented in combination with customized host cell lines and media formulations, and reported high production titers typically reflect subsequent strain, process and culture optimization rather than clonal selection alone [26, 27].

In this study, we do not aim to generate fully optimized production cell lines and processes, nor to benchmark absolute titers against industrial processes. Instead, our focus is on developing a high-throughput, secretion-based screening platform that enables rapid and reliable identification of productive antibody-producing monoclonal candidates and is broadly compatible with diverse vector systems, host cell lines, and downstream process optimization workflows.

**Fig. 2:**
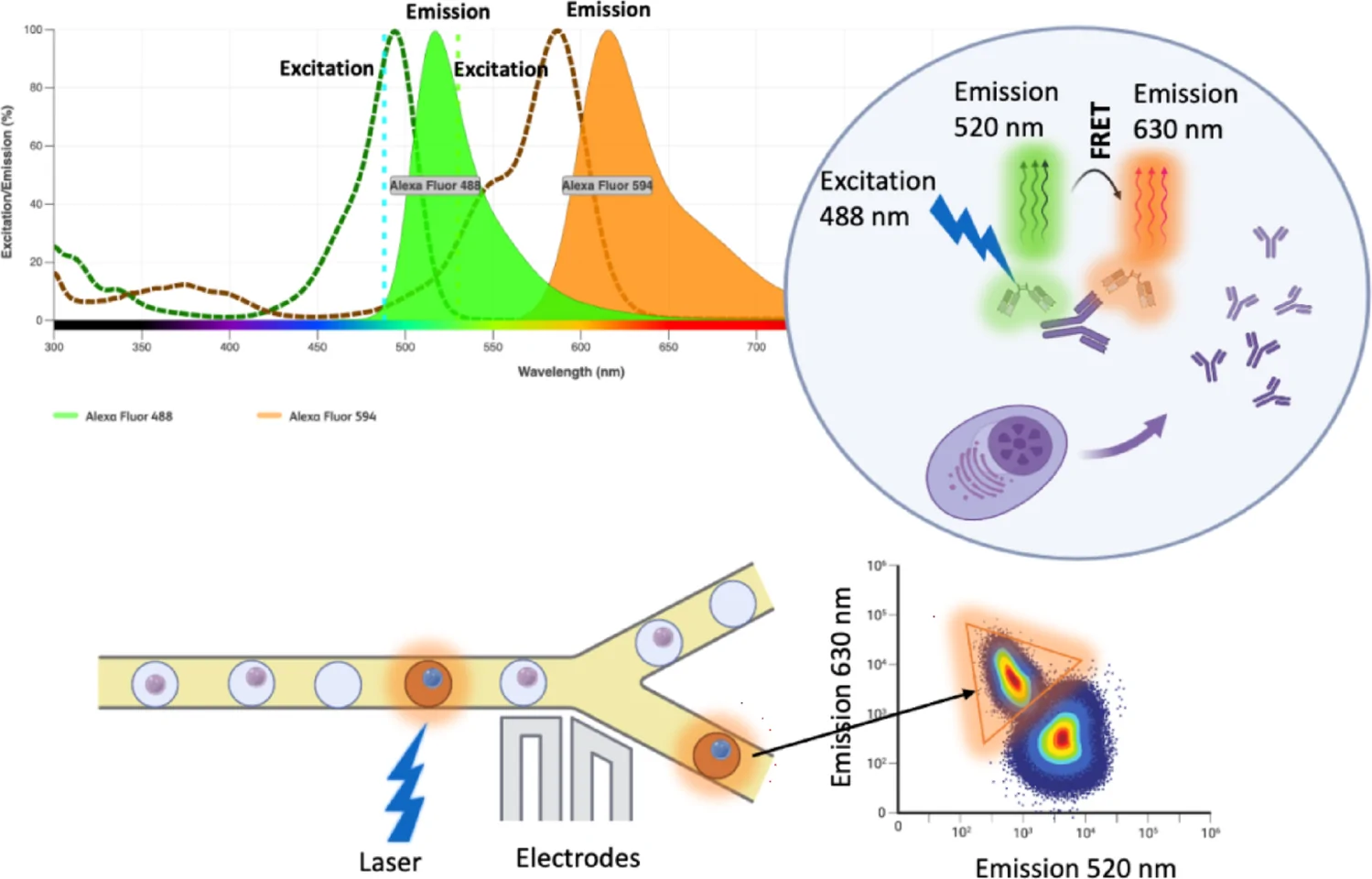
Schematic overview of the FRET-based total IgG detection assay used in this study, integrated with droplet microfluidics for high-throughput screening (created with BioRender.com). Secreted antibodies are detected using donor (488 fluorophore)- and acceptor (594 fluorophore)-labeled detection reagents that bind distinct sites on IgG molecules, producing a FRET signal upon co-binding. Individual ExpiCHO-S cells are compartmentalized into picoliter droplets containing the FRET reagents, allowing antibody secretion to accumulate locally. Droplets are optically interrogated and sorted based on FRET signal intensity, enabling discrimination and recovery of high-producing clones in a single screening run

To demonstrate the workflow, we used the pTRIOZ-mIgG1e2 expression vector, which enables coordinated heavy- and light-chain expression of a mouse IgG1 antibody under Zeocin selection in ExpiCHO-S cells. Using gevokizumab [28] an anti-IL-1β monoclonal antibody that has been clinically investigated as a representative model, we implemented a FRET-based total IgG detection assay adapted for droplet microfluidics and integrated it into an early stable CHO clone-identification workflow and monoclonal CLD process (Fig. 2).

The workflow integrates optimized donor-acceptor probe pairs with droplet-based secretion screening and automated single-cell recovery to enable rapid isolation of productive monoclonal ExpiCHO-S antibody-producing clones. Collectively, this approach compression of the early clone-identification phase relative to conventional workflows while maintaining monoclonality and scalability across antibody formats and is designed to be extendable to other secreted recombinant biologics (e.g., Fc fusion proteins, bispecific antibodies etc.) by adapting the underlying secretion-detection chemistry and calibration strategy.

## Results

### FRET-based assay development for total IgG detection

To enable high-throughput detection of antibody-secreting ExpiCHO-S cells in droplet microfluidics, we first developed and optimized a FRET-based assay for total IgG detection. Our objective was to establish a sensitive, low-background assay capable of quantifying antibody secretion in picoliter-volume droplets and discriminating high-producing cells from low or non-producers. To this end, we evaluated donor-acceptor probe pairs consisting of anti-Fc and anti-F(ab’)₂ reagents, including both full-length antibodies and F(ab’)₂ fragments, as well as Protein G- and Protein L-based binding reagents, as detailed in Table S1. All components were conjugated to FRET-compatible fluorophores, and probe combinations were assessed for their ability to form ternary complexes with mouse IgG, thereby bringing donor and acceptor fluorophores into close proximity to generate a measurable FRET signal.

**Fig. 3:**
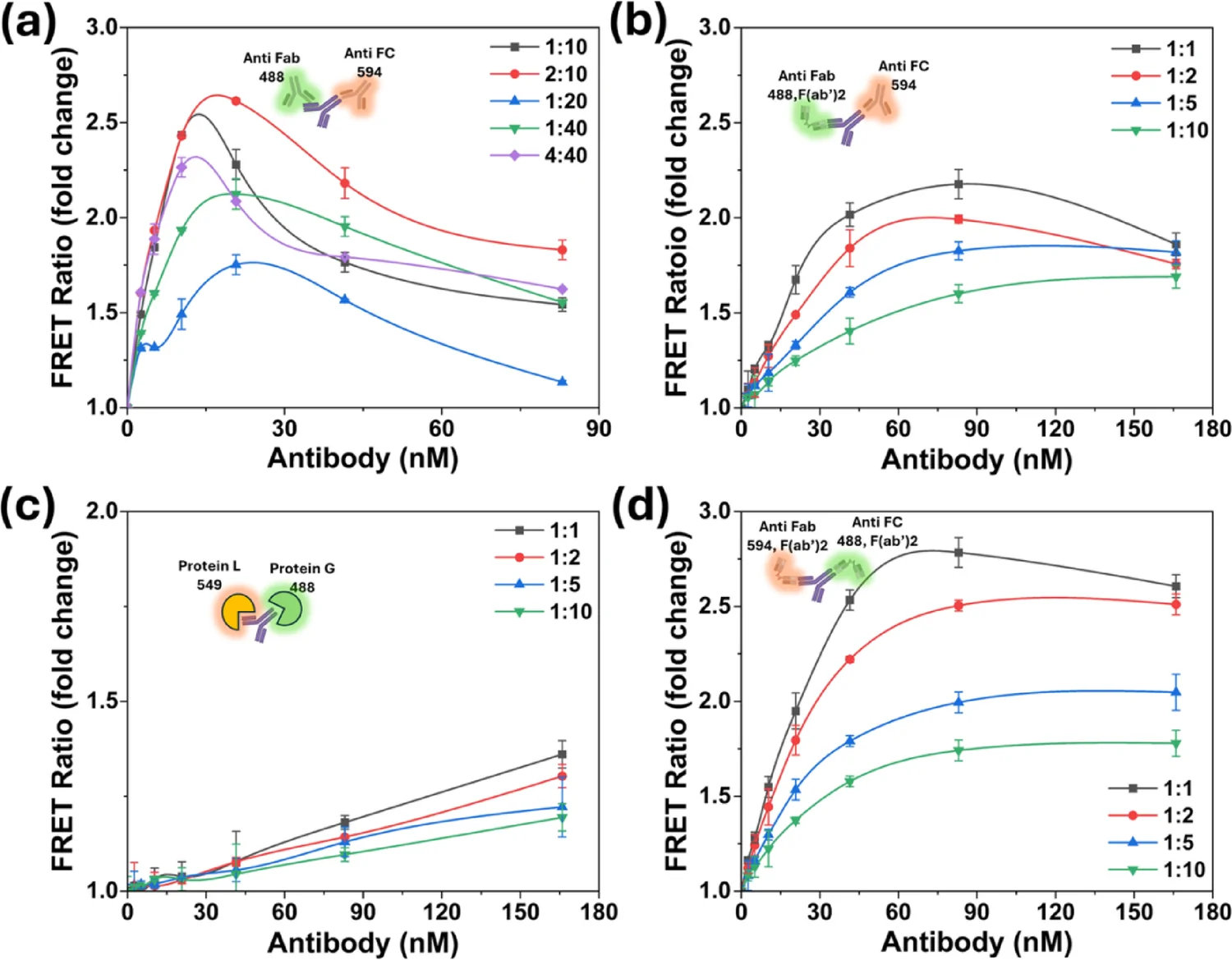
Optimization of donor-acceptor probe configurations and molar ratios for FRET-based total IgG detection. FRET performance was evaluated in microtiter plate format across a range of IgG concentrations using donor-to-acceptor ratios from 1:1 to 1:10. Panels show representative probe configurations: **a** full-length anti-F(ab’)₂ (donor) and anti-Fc (acceptor) antibodies, **b** anti-F(ab’)₂ F(ab’)₂ fragments (donor) with full-length anti-Fc antibodies (acceptor), **c** Protein G- and Protein L-based probes and **d** anti-Fc and anti-F(ab’)₂ F(ab’)₂ fragment pairs. FRET signal is reported as the emission ratio at 620 nm (acceptor) to 520 nm (donor) as a function of total IgG concentration. Data are shown as mean ± SD (*n* = 3)

Initial assay optimization and FRET pair evaluation was performed in 384-well microtiter plates using purified mouse IgG standards spanning concentrations from 0 to 166 nM. Because FRET efficiency depends on both fluorophore spectral overlap and intermolecular distance, donor-to-acceptor molar ratios were systematically optimized to enhance ternary complex formation and maximize energy transfer. Ratios ranging from 1:1 to 1:10 were evaluated for each probe combination. FRET performance was quantified by measuring emission intensity at 620 nm (FRET signal) relative to donor emission at 520 nm, and FRET efficiency was expressed as the 620/520 emission ratio. Comparative results for all probe combinations are summarized in Fig. 3.

Probe pairs composed of full-length anti-F(ab’)₂ (donor) and anti-Fc (acceptor) antibodies produced a maximum FRET signal of approximately 2.5-fold at an IgG concentration of 20.75 nM (~ 3.1 mg/L) when tested at donor-to-acceptor ratios of 1:5 and 1:10; however, a hook effect was observed at higher antibody concentrations, resulting in decreased FRET signal intensity (Fig. 3a). As a consequence, low- or non-producing cells and high-producing cells can generate comparable FRET signals, severely limiting the assay’s ability to reliably rank secretion levels and discriminate top producers during droplet-based sorting.

A second probe pair consisting of an anti-F(ab’)₂ F(ab’)₂ fragment (donor) and a full-length anti-Fc antibody (acceptor) generated a comparable but similarly constrained FRET response (~ 2-fold) at IgG concentrations of 41.5 and 83 nM when tested at donor-to-acceptor ratios of 1:1 and 1:2 (Fig. 3b), indicating a limited operational window for reliable secretion quantification. In contrast, the Protein G (donor) and Protein L (acceptor) probe pair did not yield a meaningful FRET response (< 1.5-fold) across all IgG concentrations tested, including concentrations up to 166 nM (Fig. 3c), suggesting an inefficient proximity-driven energy transfer under these conditions.

Notably, the probe pair composed of anti-Fc F(ab’)₂ fragments (donor) and anti-F(ab’)₂ F(ab’)₂ fragments (acceptor) produced a strong and sustained FRET response, exceeding 2-fold across a broad IgG concentration range (41.5–166 nM) when tested at donor-to-acceptor ratios of 1:1 and 1:2, with the 1:1 ratio yielding the highest and most stable signal (> 2.5-fold) throughout the tested range (Fig. 3d). Based on its superior signal stability, dynamic range, and robustness across donor-acceptor ratios, this probe configuration was selected for subsequent implementation in droplet microfluidic single-cell antibody secretion detection assays.

### FRET-based picodroplet detection and sorting of defined ExpiCHO cell mixtures

To evaluate the performance of the optimized FRET-based total IgG detection assay in a screening context, we generated an artificial cell library by mixing defined ratios of antibody-secreting and non-secreting cells, mimicking the heterogeneity typically observed in early stages of stable CLD. ExpiCHO-S cells were transiently transfected with the circular pTRIOZ-mIgG1e2 plasmid encoding both the heavy and light chains of gevokizumab. Gevokizumab-transfected cells were labeled with CellTrace™ Far Red (CTFR) to enable downstream flow-cytometric discrimination without interfering with the donor or acceptor fluorophores used in the FRET assay during droplet microfluidic detection and sorting. Transfected cells (5%) were then mixed with non-transfected ExpiCHO-S cells (95%), which served as the non-producing population.

**Fig. 4:**
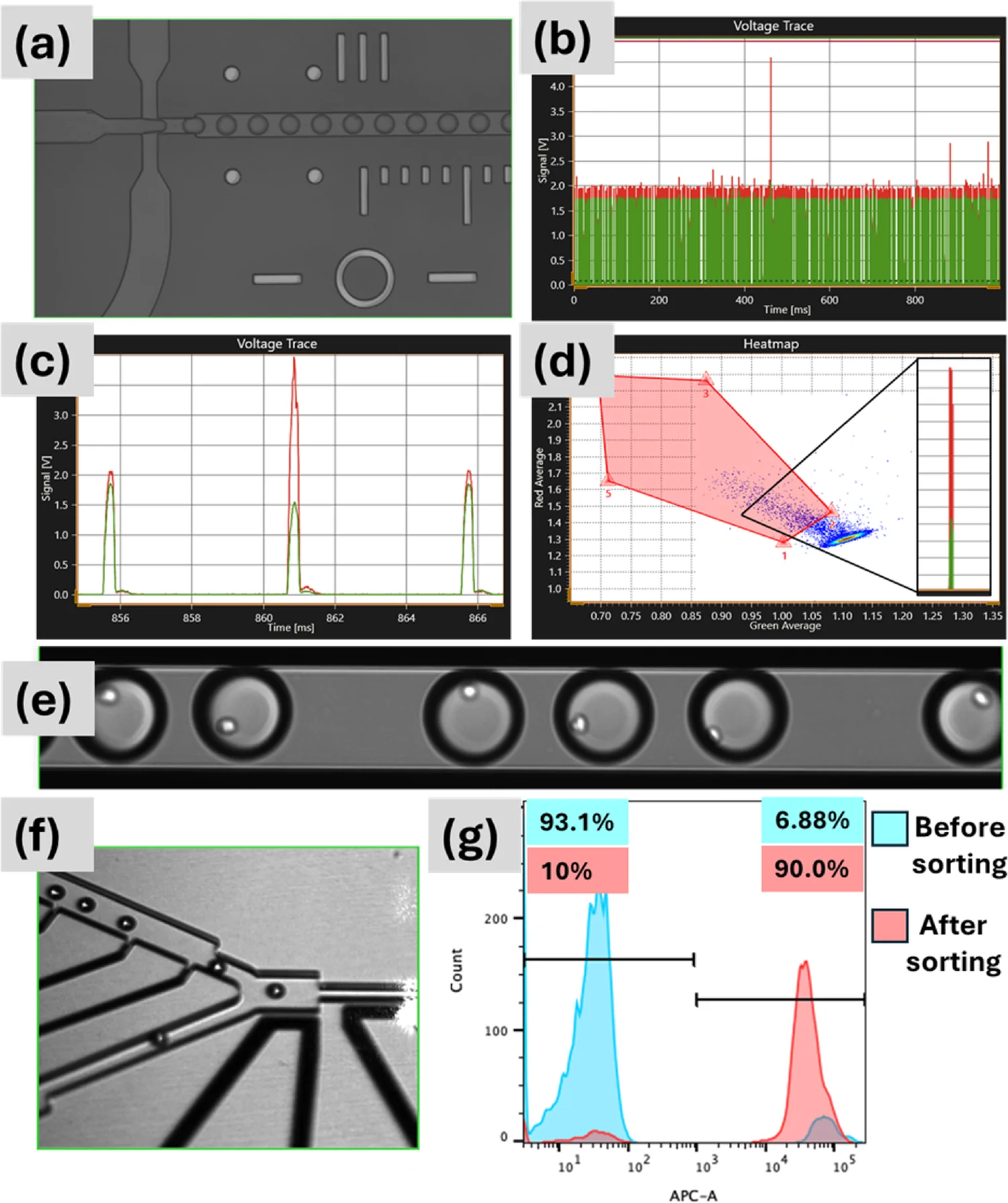
Droplet microfluidics platform integrating picodroplet encapsulation, secretion-based screening, sorting, dispensing, and image-based monoclonality assessment (**a**-**g**). **a** Encapsulation of individual ExpiCHO-S cells into picoliter droplets, **b** Detection of donor fluorescence (green) and FRET-induced acceptor emission (red), used for quantifying secreted antibody within droplets, **c** Representative voltage traces showing donor and FRET-channel signal intensities during droplet interrogation, **d** FRET scatterplot illustrating gating of droplets exhibiting elevated acceptor-to-donor fluorescence ratios, **e** Images of picodroplets following sorting, **f** Sorting of gated picodroplets into the collection channel, **g** Flow cytometry analysis of the artificial mixed library before and after sorting, demonstrating enrichment of the CTFR-positive (APC-channel) antibody-secreting population

The mixed cell suspension was co-encapsulated into picodroplets (Fig. 4a) along with the optimized FRET detection reagents-anti-Fc F(ab’)₂ (donor) and anti-Fab F(ab’)₂ (acceptor) at a 1:1 ratio, and incubated at 37 °C for 60 min to allow antibody accumulation. Cell loading into picodroplets followed Poisson statistics, resulting predominantly in droplets containing either a single cell or no cell, with a smaller fraction containing multiple cells. Within each picodroplet, secreted antibody generated a measurable FRET signal, reflected by increased acceptor fluorescence with a corresponding decrease in donor emission (Fig. 4b). Picodroplets exhibiting high acceptor-to-donor ratios-located in the upper-left region of the scatter plot (Fig. 4d) were gated, sorted and pooled-dispensed for recovery (Fig. 4e). The cluster in the lower-right region represents the majority of events, corresponding to picodroplets containing low- or nonproducing cells as well as empty droplets (Fig. 4d).

After sorting and pooled dispensing, picodroplets were demulsified using PicoBreak™ to recover the encapsulated cells, and enrichment of antibody-secreting cells was quantified by flow cytometry using the CTFR dye. Prior to sorting, 6.88% of the artificial mixed library fell within the CTFR-positive gate, consistent with the intended ~ 5% proportion of producers. Following a single round of picodroplet sorting, the recovered population consisted of approximately 90% CTFR-positive cells and 10% unstained cells. Thus, the initial 5% producer population was enriched to ~ 90% (Fig. 4g), demonstrating that the optimized FRET assay and Cyto-Mine^®^ workflow effectively identifies and isolates antibody-secreting cells from a heterogeneous mixture containing predominantly non-producers. Individual scatter plots of the CTFR-stained and unstained cell populations prior to mixing, as well as the mixed library before and after sorting, are provided in Supplementary **Figure S1**. Consistent enrichment was observed across independent artificial-library sorting experiments. Libraries containing approximately 5–10% producer cells were consistently enriched to approximately 90–93% producer cells after FRET-based droplet sorting, supporting reproducible enrichment of antibody-secreting cells under the optimized assay condition.

### Culture medium optimization for efficient single-cell clonal expansion

Following FRET-based droplet sorting, the ability of individual ExpiCHO-S cells to survive and expand is essential for establishing monoclonal stable cell lines. To determine the most suitable conditions for single-cell outgrowth, we evaluated three media formulations: ECEM (ExpiCHO Expression Medium); ECEM supplemented with HT (hypoxanthine-thymidine); and LCD medium, consisting of 50% conditioned ECEM (supernatant from non-transfected ExpiCHO-S cultures), 47% fresh ECEM, 2% glutamine, and 1% HT supplement. Single cells were deposited into 96-well plates using a FACSAria™ Fusion sorter, and colony formation was assessed on Day 14. Among the tested formulations, ECEM yielded the most consistent and highest single-cell outgrowth (30–40%), whereas ECEM + HT and LCD medium supported markedly fewer surviving colonies (Supplementary Figure S2). These results indicate that post-sorting recovery is highly dependent on medium composition, and that for this particular cell line, ECEM provides the most favorable environment for single-cell survival under our workflow conditions. Based on these findings, ECEM was chosen as the medium for Cyto-Mine^®^ single-cell deposition in the subsequent stable cell line generation experiments.

### Stable cell line sorting

Following optimization of the FRET-based IgG detection assay and identification of optimal single-cell survival conditions, we applied the complete workflow to generate stable CHO cell lines producing the monoclonal antibody gevokizumab. ExpiCHO-S cells were transfected with the linearized pTRIOZ-mIgG1e2 vector, which encodes both antibody chains under Zeocin selection. Antibiotic titration in ExpiCHO-S cells identified 50 µg/mL zeocin as an appropriate selection concentration for the experimental selection window (Supplementary Figure S3), consistent with the manufacturer-recommended stable-pool generation strategy, in which the ideal selective pressure kills approximately 50% of cells and/or inhibits growth around days 3–5. This concentration provided substantial selection pressure while maintaining sufficient viable transfected cells for subsequent FRET-based enrichment and was therefore used throughout the workflow. This concentration was subsequently used to enrich the transfected CHO cell population prior to FRET-based screening. Unlike conventional CLD workflows, where stringent antibiotic selection is used to establish a stable cell pool before clone screening, our strategy combines a shorter, less stringent selection step with functional FRET-based enrichment. Zeocin selection was maintained while FRET-based sorting was used as an early antibody-expression enrichment step to remove non-expressing or poorly expressing cells before single-cell isolation. At Day 6 post-transfection, after approximately 4 days of Zeocin selection, an initial FRET-based bulk sorting round was applied. Each cell was encapsulated into a picodroplet containing the optimized FRET detection reagents and incubated for 60 min to allow antibody accumulation and signal development. Droplets exhibiting elevated FRET signals indicative of antibody secretion were sorted, pooled-dispensed (~ 4,000 droplets), and expanded in ECEM supplemented with Zeocin. Although direct single-cell deposition after early selection is possible in principle, this intermediate enrichment was necessary because the initial viability frequency of antibody-producing cells in the transfected pool was low (0.1–1%), and direct single-cell sorting at this stage would have yielded very few productive clones. Bulk enrichment therefore provided a more robust starting population for subsequent monoclonal isolation but final clone identification was based on clonal recovery, expansion, and maintenance of antibody production over extended culture, including culture in the absence of selection pressure.

**Fig. 5:**
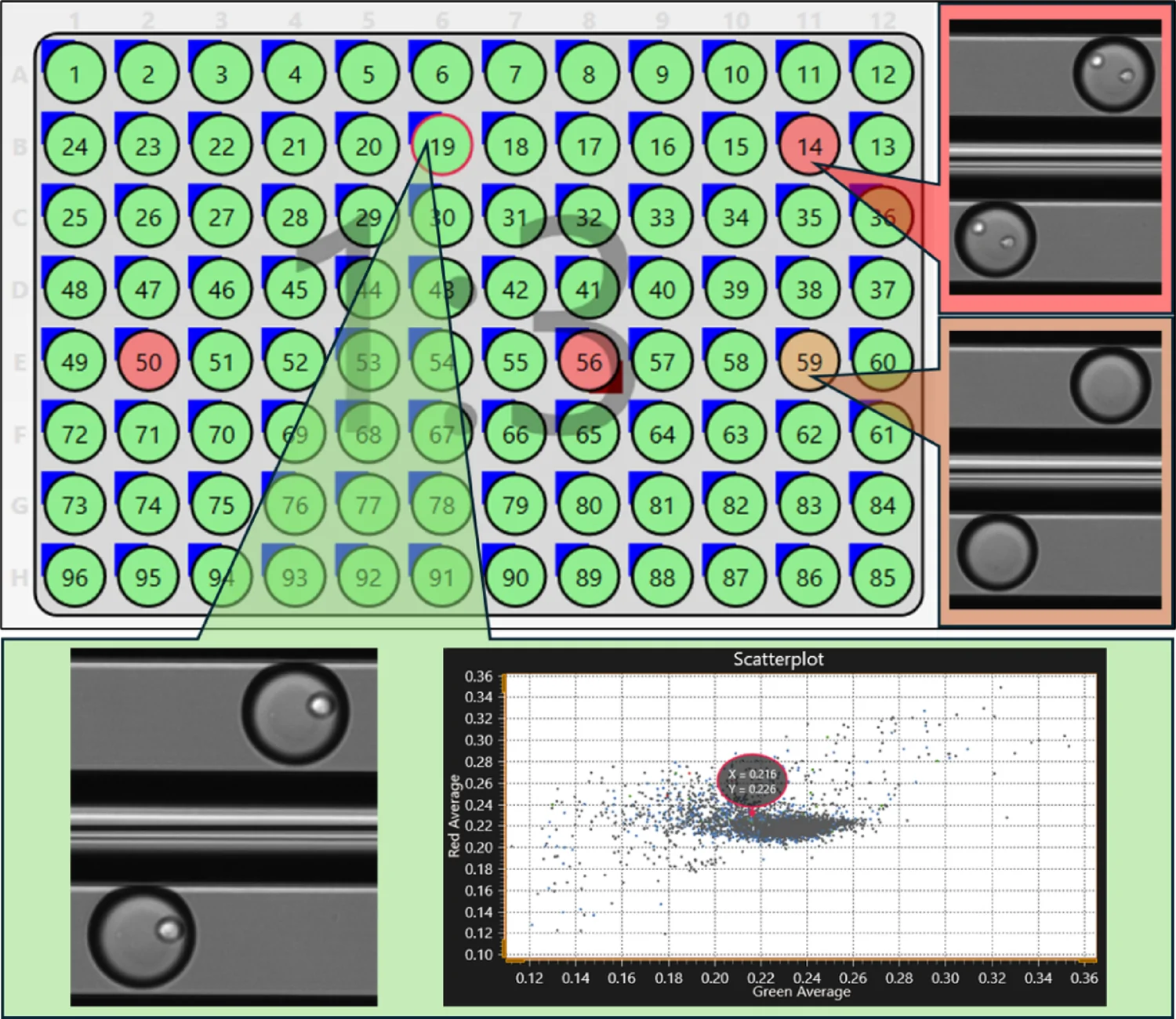
Post-sorting picodroplet imaging and monoclonality verification following picodroplet-based deposition into 96-well plates. Cyto-Mine^®^ image-based single-cell occupancy assessment is achieved through automated picodroplet imaging: each droplet is captured in five sequential brightfield images (two representative images shown) immediately prior to deposition, enabling retrospective verification of monoclonality. Wells are color-coded according to the image-based classification: green indicates single-cell deposition, red indicates picodroplets containing multiple cells, and yellow denotes ambiguous cases where monoclonality cannot be confidently confirmed. Representative images illustrate picodroplets containing zero, one, or two cells. The accompanying scatterplot shows the position of the corresponding picodroplet within the gated and sorted population

Preliminary experiments using non-transfected CHO cells, the picodroplet sorting and demulsification procedure resulted in approximately 89% cell recovery and 81% immediate post-recovery viability (data not shown), consistent with previous reports demonstrating > 80% CHO cell viability following droplet encapsulation and subsequent emulsion breaking [11, 29]. After 14 days of recovery and expansion under Zeocin selection, the enriched stable pool exhibited approximately 94% viability and an antibody titer of 11.0 mg/L before undergoing a second sorting round in which individual antibody-secreting cells were isolated. Thus, by the time of single-cell deposition, the sorted cells had undergone approximately three weeks of post-transfection Zeocin selection. Non-transfected control cells did not remain viable under comparable Zeocin selection conditions, supporting that survival at this stage depended on Zeocin resistance, consistent with stable integration of the selection cassette. Droplets with the highest FRET intensities were dispensed as single droplets into five 96-well plates to enable outgrowth of monoclonal lines. During deposition, the Cyto-Mine^®^ system captures both fluorescence outputs and brightfield images for monoclonality assessment. For each droplet, five sequential images were acquired immediately upstream of the dispensing nozzle (Supplementary Figure S4), allowing visualization of cell number and relative movement. These images, together with fluorescence readouts and well addresses, were used to verify monoclonality. Representative examples of droplets containing two cells, an empty droplet, and a single cell are shown in Fig. 5.

Of the 480 droplets dispensed, 6.67% were identified by the five-frame Cyto-Mine^®^ imaging as containing ≥ 2 cells, and 2.29% were identified as empty. These events were recognized during image-based quality control and were excluded from downstream clone selection. The remaining wells contained single-cell-derived colonies, of which more than 250 recovered after sorting. From these, 184 image-verified monoclonal populations were selected for antibody production analysis 7 days post-sorting using ELISA, ensuring that only confirmed single-cell-origin clones were carried forward for characterization (Supplementary Figure S5). This initial ELISA screen was used to rank recovered clones based on total antibody accumulation in 96-well culture after the same recovery period, thereby capturing both antibody secretion and clonal recovery/growth. Clone-specific productivity (qP) was subsequently determined after expansion for selected representative clones.

### Antibody productivity evaluation and stable cell line generation

**Fig. 6:**
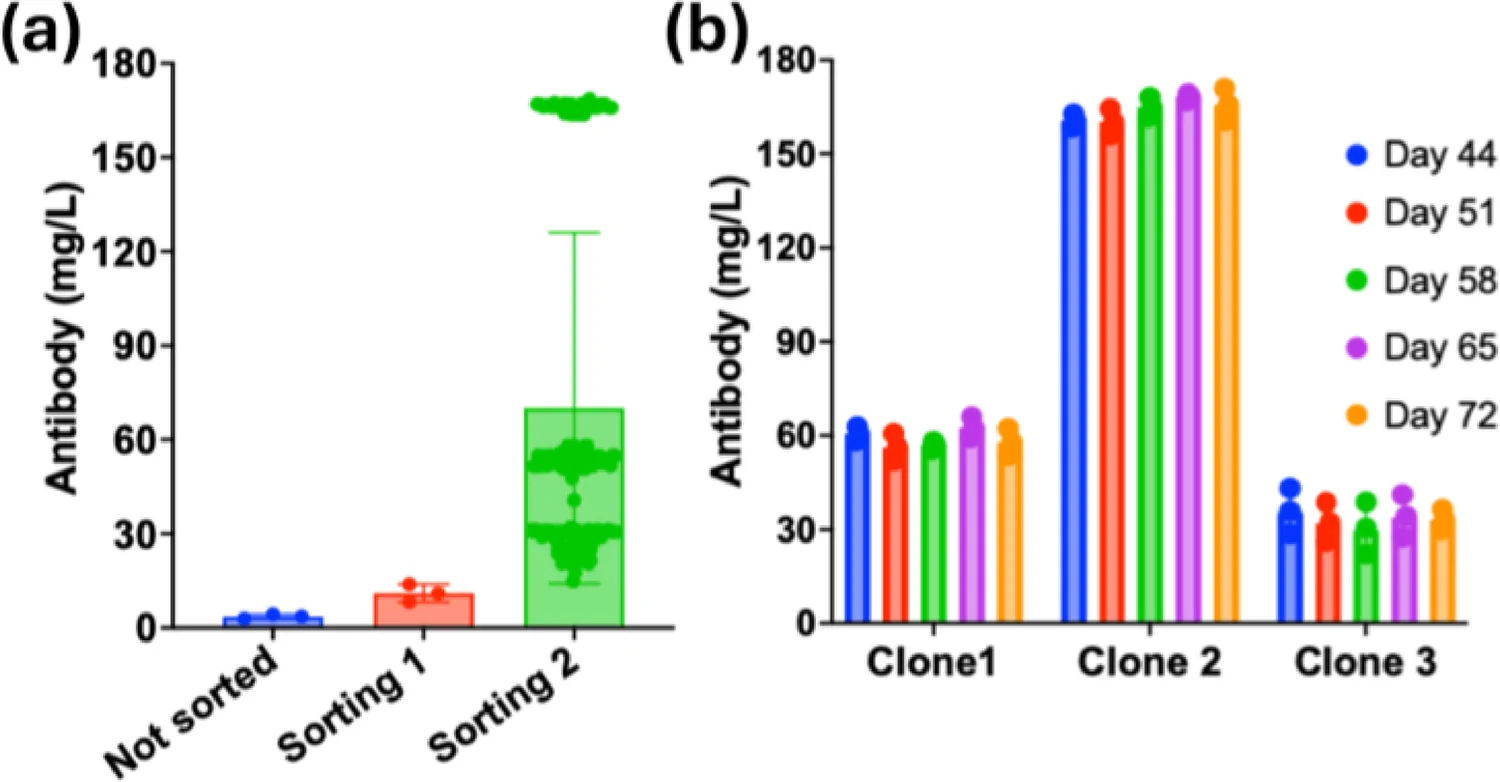
**a** Total antibody titer measured by ELISA at successive stages of the workflow, showing increased gevokizumab production following stable transfection, bulk FRET-based enrichment, and monoclonal clone isolation. **b** Specific productivity of three selected monoclonal ExpiCHO-S cell lines measured over a 90-hour assay following medium exchange and tracked across multiple passages. Data are presented as mean ± standard deviation (*n* = 3)

Total antibody titer increased progressively throughout the workflow (Fig. 6a). Following stable transfection, the initial polyclonal pool produced 3.6 mg/L of gevokizumab. After the first, bulk FRET-based sorting step, antibody titer increased to 11.0 mg/L. Following the second round of sorting and single-cell isolation, antibody production further increased, with an estimated average titer of ~ 80 mg/L based on ELISA measurements of individual monoclonal cultures.

After 14 days of post-sorting recovery, single-cell outgrowth, and ELISA confirmation, three stable ExpiCHO-S antibody producing clones were selected for further characterization. These clones were chosen to represent a range of production phenotypes, including two higher-producing clones and one clone with moderate productivity, enabling evaluation of the workflow across different secretion levels. Specific productivities were quantified after a 90-hour growth following medium exchange. Clone 3 exhibited a moderate productivity of 12.5 pg/cell/day, whereas clones 1 and 2 displayed substantially higher productivities of 34.7 and 41.3 pg/cell/day, respectively. These values are consistent with typical early-stage CHO production clones prior to process optimization [26].

Stability testing demonstrated that antibody production remained consistent over a 30-day continuous culture period (72 days post-transfection) without selection pressure, corresponding to approximately 10 passages following final clone confirmation and expansion into 25 mL shake-flask cultures. No significant decline in productivity was observed during this period (Fig. 6b), indicating stable long-term expression.

Together, this integrated workflow combining picodroplet-based secretion screening, bulk enrichment, image-verified single-cell isolation, and efficient clone expansion reduced the early CLD timeline from DNA transfection to image-verified, antibody-positive 96-well clone candidates in approximately 35–37 days, compared with the 16 weeks typically required using traditional limiting dilution cloning. This streamlined approach provides a robust and broadly adaptable framework for generating stable CHO producers of monoclonal antibodies and other therapeutic protein formats.

## Discussion and conclusions

The prolonged timelines associated with conventional antibody-producing CLD arise not only from antibiotic selection, but also from the need for repeated rounds of clonal isolation to ensure monoclonality, followed by extensive clone expansion, productivity screening, and stability assessment. A major limitation in conventional stable CHO CLD is the disconnect between clonality assurance and early productivity assessment. In standard workflows, monoclonality is often established before secretion phenotype can be directly evaluated, resulting in extended expansion and secondary screening steps before productive clones can be identified. The workflow presented here addresses this limitation by linking secretion-based screening with monoclonal recovery at the earliest stages of CLD (early enrichment of antigen-expressing cells with single-cell sorting), enabling more efficient identification of productive monoclonal CHO cell lines.

Droplet microfluidics has been broadly applied to preserve genotype-phenotype linkage in high-throughput screening workflows, including secretion-based protein detection and enzyme activity assays [9, 10, 30, 31]. In the specific context of stable CHO CLD, this principle enables early secretion-linked identification of productive clones while maintaining single-cell compartmentalization for downstream monoclonal recovery. The workflow presented here applies this concept to address a key limitation in conventional CLD by linking secretion phenotype with monoclonal recovery at the earliest stages of clone selection, while providing a practical framework for implementing secretion-based screening in routine CHO CLD.

Despite growing interest in droplet-based microfluidic approaches for antibody screening, most published workflows remain focused on proof-of-concept demonstrations or target-specific discovery applications [15, 16, 31] and provide limited guidance on assay optimization, post-sort recovery, and integration into stable CHO CLD. As a result, reproducible and transferable workflows for secretion-based droplet screening in routine CLD remain limited.

In this study, we addressed this gap by systematically developing and optimizing a total IgG FRET-based secretion assay and integrating it into a picodroplet microfluidic workflow for high-throughput screening of antibody-expressing ExpiCHO-S cells. The main contribution of the present study is not the microfluidic platform itself, but the experimentally defined assay-development and workflow-integration steps required to adapt FRET-based droplet screening to early stable CHO cell line development.

Because FRET efficiency depends critically on donor-acceptor proximity (typically < 10 nm) and stoichiometry [32], we evaluated multiple detection reagent combinations and donor-to-acceptor ratios to maximize signal intensity while minimizing background. Rapid antibody accumulation within ~ 450-pL droplets can shift probe-to-analyte stoichiometry and produce signal compression or hook-like behavior, potentially causing high-producing cells to overlap with low- or non-producing populations. We therefore systematically evaluated probe format, binding configuration, and donor-to-acceptor stoichiometry rather than treating the FRET detection chemistry as a predefined component of the microfluidic workflow. Importantly, the picodroplet-based assay described here is designed to prioritize secretion phenotype at the single-cell level, which is most directly related to cell-specific productivity (qP; typically reported in pg/cell/day). In contrast, harvest titer (g/L) at manufacturing scale is an integrated outcome of qP multiplied by the integrated viable cell density over the duration of the culture, and is therefore strongly influenced by multiple up- and downstream process parameters. These include improved medium and feed composition, culture mode (batch, fed-batch, or perfusion), bioreactor operating conditions, viable cell titers and overall process duration. Consequently, volumetric titer cannot be considered a clone-only attribute, but rather the result of coordinated clone selection and subsequent strain and process optimization [26]. This distinction is essential when interpreting “high producer” claims reported across different workflows and timescales and is consistent with contemporary biomanufacturing literature emphasizing that early-stage clone screening should focus on robust secretion phenotypes, with final titers realized through later-stage media, cell line and process development steps that are beyond the scope of the present study.

Assay design was informed by reported secretion rates for antibody-producing cells, which range from ~ 200 IgG molecules per second per cell for hybridomas to ~ 2,000 molecules per second per cell for plasma B cells [31, 33]. When combined with picoliter-scale droplets (~ 450 pL), these secretion rates correspond to low-nanomolar to tens-of-nanomolar range (approximately ~ 1–30 nM) after 30–60 min of incubation, matching the concentration window used for construction of the FRET assay standard curve.

Probe format and donor: acceptor ratio strongly influenced assay performance and dynamic range, making empirical optimization essential for reliable secretion-based screening. Among the configurations tested, the Fab’₂/Fab’₂ anti-Fc and anti-F(ab’)₂ probe pair at a 1:1 ratio produced the strongest and most stable response across the relevant IgG concentration range (Fig. 3c), enabling robust discrimination of antibody-secreting cells. In contrast, Protein G/Protein L-based detection-previously mentioned as a platform capability [34] did not produce comparably robust FRET responses under our conditions (Fig. 3c), underscoring that secretion assays remain highly dependent on probe chemistry and assay configuration.

Although signal saturation was observed at higher antibody concentrations, this limitation can be managed at the workflow level by reducing droplet incubation time or applying sequential sorting rounds to maintain measurements within the linear dynamic range. From a practical CLD perspective, this is particularly important because insufficient assay optimization can lead to false ranking of productive clones through signal compression, probe competition, or hook-like effects during the earliest and most time-sensitive stages of screening [32, 35, 36].

These results emphasize that assay optimization should be performed in microtiter plate format using the specific reagents available in each laboratory before transfer into droplet workflows. By explicitly outlining this optimization strategy, the present study provides a practical framework for implementing secretion-based FRET screening in CHO CLD and related secretion-driven workflows.

To model the heterogeneity typical of early-stage stable CLD, we generated synthetic ExpiCHO-S libraries containing a low frequency of antibody-secreting cells. This reflects real-world stable transfection pools, in which productive cells are rare and must be separated from a large background of non-producers. Using optimized FRET reagents, we demonstrated efficient enrichment of antibody-secreting cells following droplet-based sorting. Unlike conventional FACS-based strategies, which often rely on indirect markers such as surface staining or reporter expression [37], the droplet-based assay directly quantifies secreted antibody within a confined microenvironment, preserving the genotype-phenotype link and enabling accurate recovery of antibody-producing cells.

Droplet encapsulation inherently follows Poisson statistics, and therefore absolute avoidance of multi-cell droplets is not possible when targeting single-cell occupancy. Importantly, in the present workflow monoclonality is not assumed based on Poisson distribution alone. Instead, single-cell occupancy was assessed by image-based quality control at the point of droplet deposition. Automated brightfield imaging of each droplet immediately prior to dispensing enables retrospective determination of cell number, and only image-verified single-cell droplets are advanced for downstream analysis. This two-layer approach statistical enrichment combined with direct visual confirmation addresses a key limitation of many CLD workflows, in which monoclonality is inferred but not experimentally confirmed. Notably, the deposition-imaging step provides an operational and documentable mechanism to exclude multi-cell events during clone recovery, which is central to the credibility of “monoclonal” cell line claims in CLD workflows. In the present study, monoclonality verification is performed at the same step as recovery (at deposition), which eliminates the need to infer monoclonality indirectly from loading statistics.

The reliability of this approach has previously been characterized in CHO cells by Josephides et al., who reported 98.93% agreement between the cell number identified by pre-dispense Cyto-Mine imaging and the actual number of cells observed after deposition [19]. Pybus et al. subsequently used the same five-frame Cyto-Mine imaging approach as part of a multi-stage monoclonality assurance framework and estimated the probability of Cyto-Mine dispensing error, P(d), at 0.05% [20]. Together, these studies support the reliability of the five-frame Cyto-Mine assessment for image-based verification of single-cell occupancy. If required for regulatory compliance or for applications requiring a formally calculated probability of monoclonality, additional quality-control steps can be incorporated into the workflow, including orthogonal post-deposition plate imaging and probability-of-monoclonality analysis as described by Pybus et al.

More broadly, comparison of monoclonality workflows and CLD timelines is complicated by the absence of a single universally mandated benchmark defining the required assay, instrument, cloning technology, number of cloning rounds, probability threshold, or duration of stability monitoring across all recombinant CHO cell line development programs. Regulatory guidance instead emphasizes clonal derivation and appropriate characterization of the production cell substrate and cell bank system, with expectations interpreted in the context of the intended product, process, and control strategy. Therefore, the endpoint and duration of a CLD workflow depend on clone recovery and growth, expression stability, cell-banking strategy, downstream process requirements, and product-quality expectations. In this context, the 35–37-day timeline reported here should be interpreted as the early clone-identification phase from DNA transfection to validated 96-well antibody-producing clones, rather than completion of all downstream cell banking, extended stability assessment, process development, and regulatory characterization activities.

Single-cell recovery and outgrowth represent additional bottlenecks in CLD workflows. Post-sort survival can be limited by mechanical stress, emulsion breaking, and the loss of paracrine support present in bulk cultures [38]. While conditioned media and proprietary additives can enhance survival [32–34], these approaches introduce variability and are not always chemically defined. In contrast, the chemically defined medium used here provided consistent single-cell outgrowth while minimizing lot-to-lot variability and regulatory risk. Moreover, the droplet shell itself acts as a protective microenvironment that mitigates shear stress and cross-contamination during sorting and handling [42, 43]. Because recovery is often an underappreciated determinant of “effective throughput,” improving post-sort survival directly contributes to the real-world speed of obtaining enough confirmed monoclonal cell line producers for downstream ranking and stability assessment.

A key conceptual distinction between the workflow presented here and classical CLD strategies lies in the timing of selection. Traditional CLD relies on prolonged antibiotic selection over weeks to months, under the implicit assumption that stable integration emerges gradually over time. In reality, genomic integration events occur almost immediately following transfection; extended selection periods primarily serve to allow non-integrated or non-expressing cells to be eliminated. In the workflow described here, secretion-based sorting is performed early, enabling rapid isolation of all cells actively expressing the recombinant antibody whether expression is transient or stably integrated. Direct single-cell deposition immediately after transfection and brief antibiotic selection is possible however may be inefficient when the frequency of FRET-positive cells is very low, because only a limited number of droplets can be practically screened and imaged during a sorting run. Under these conditions, an initial bulk enrichment step increases the proportion of antibody-secreting cells before image-verified single-cell deposition.

Bulk enrichment at this stage captures the full expressing population, while subsequent recovery and expansion allow transient expression to decay. A second round of secretion-based sorting performed several days later selectively isolates cells that continue to express antibody, thereby enriching for stably integrated clones. This strategy decouples expression screening from prolonged antibiotic selection and accelerates the path to monoclonal, stable producers. From a “speed of high-producer identification” perspective, the key practical benefit is that secretion phenotype is measured and acted upon early, rather than waiting for weeks or months of antibiotic selection and expansion before productivity ranking can begin. The timeline reported here should be interpreted as an early clone-identification timeline for the specific host cell line, selection conditions, and antibody model used in this study. In practice, CLD timelines are influenced by cell viability after transfection and antibiotic selection, recovery after sorting, growth rate during expansion, and the extent of stability monitoring required for a given product or application. For example, some enriched pools or single-cell-derived cultures may recover within approximately one week, whereas others may require longer recovery before sufficient cell numbers are available for the next workflow step. Similarly, the number and timing of enrichment rounds can be adjusted depending on the frequency of FRET-positive cells, post-selection viability, and the desired number of recovered clones. Thus, the workflow is best viewed as a flexible droplet microfluidics-assisted CLD strategy rather than a rigid fixed-day protocol.

The monoclonal ExpiCHO-S cell lines generated using this approach exhibited stable antibody expression and productivities consistent with early-stage production clones. While anecdotal reports in the literature cite antibody titers exceeding several grams per liter, such values are typically achieved only after extensive strain improvement including gene amplification, media optimization, bioreactor and process development, and are rarely documented with sufficient methodological detail to enable direct comparison [37–41]. The objective of the present study was not to maximize absolute titer, but to establish a rapid, reproducible screening and monoclonality-assessment workflows for stable CLD. Additional up- and downstream process optimization steps can be applied following initial clone selection. Consistent with this “fit-for-purpose CLD” positioning, the workflow increased total antibody titer stepwise from the initial polyclonal pool (3.6 mg/L) to post-enrichment (11.0 mg/L) and to approximately ~ 80 mg/L following the second sorting and monoclonal isolation stage. Although more than 200 image-verified monoclonal populations were recovered after single-cell sorting, a small subset of clones was selected for detailed characterization. From these, three representative monoclonal ExpiCHO-S cell lines were expanded and analyzed in depth, exhibiting quantified specific productivities of 34.7 (clone 1), 41.3 (clone 2), and 12.5 (clone 3) pg/cell/day (Supplementary Figure S6a). Whereas total antibody production of these clones was determined as 58 mg/L, 166 mg/L, and 32 mg/L, respectively (Supplementary Figure S6b). The difference between volumetric titer and qP reflects that these metrics capture different aspects of clone performance. While volumetric titer represents the total antibody accumulated in culture, qP is normalized to viable cell density and culture duration. Therefore, differences in cell growth and viable cell density during the production phase can contribute to differences between these measurements. The droplet FRET signal was used as a threshold-based secretion-enrichment criterion rather than as a calibrated predictor of clone-specific productivity. Sorted wells could be traced back to FRET-positive events, supporting functional enrichment of antibody-secreting cells; however, direct quantitative correlation of initial droplet FRET intensity with qP would require clone-matched fluorescence-intensity values and subsequent qP calculation for each recovered clone.

These qP values fall in the “tens of pg/cell/day” category commonly associated with strong early-stage producers prior to extensive strain and process optimization [26]. A potential concern with our early enrichment strategies is the isolation of transiently expressing cells. In our workflow, bulk-sorted cells were cultured for an additional 14 days before single-cell cloning to reduce this possibility. Moreover, the selected clones maintained stable antibody production from Day 44 to Day 72 after transfection (approximately 10 passages after clone isolation), with no detectable decline in productivity (Fig. 6b). These findings support that the two-step sorting strategy effectively identified stable producer clones. Therefore, this study demonstrates stability of antibody production over a 30-day continuous culture period (72 days post-transfection) in the absence of antibiotic selection pressure, supporting the conclusion that early secretion-based selection can converge on genuinely stable, antibody-producing clones rather than predominantly transient expressors.

In comparison with previously reported CHO CLD strategies (Table 1), the workflow described here achieves comparable early-stage titers while substantially reducing development timelines from months to approximately 5–8 weeks and providing explicit monoclonality verification. By focusing on assay development, secretion-based screening, and post-sort recovery, this work complements existing bioprocess optimization approaches rather than replacing them. In particular, Table 1 places the present study’s early-stage titers (32–166 mg/L) and timeline (5 weeks) in direct context with commonly cited approaches such as MTX-based amplification, ClonePix-based selection, and pool-based BAC workflows, reinforcing that the principal advantage here is speed with monoclonality assurance rather than claiming industrial-scale titers at screening stage. The antibody titers reported here should be interpreted as those of unoptimized early-stage lead clones rather than fully optimized industrial production cell lines. Further increases in productivity would likely require additional optimization steps, including vector design, gene amplification strategies such as DHFR/MTX selection, media and feed optimization, and fermentation/process development.

**Table 1:** Comparative summary of reported strategies for antibody-producing stable CHO cell line development, including cell line type, culture media, expression vector, development approach, antibody titer, and development timeline

Cell line	Media/selection agent	Expressed antibody	Vector	Approaches (Transfection/Single-cell-clone)	Antibody titer (mg/L)	Timeline (Days)	References
CHO DG44	HyQ PF-CHO and CD CHO/MTX	IgG	phCMV-VHRhD-g1C-neo and phCMV-VLRhD-KR -neo	Electroporation/LDC	120–220	ND	[44]
CHO DG44	CD OptiCHO/MTX	IgG	pOptiVEC-TOPO	Lipofectamine/ClonePix FL	40–42	ND	[45]
CHO-DG44 and CHO-S	CD OptiCHO/MTX	IgG	pcDNA3.3-TOPO and pOptiVEC-TOPO	FreeStyle MAX/LDC	1000	ND	[46]
CHO-K1, CHO-DG44, and CHO-S	CD CHO and ActiCHO P/-	IgG (from stable pool)	BAC	ND/ND	55–390	ND	[47]
CHO-S	CD CHO and CD OptiCHO/-	IgG (from stable pool)	pCI	Electroporation (MaxCyte STX)/ND	1000	ND	[48]
CHO-S	ExpiCHO/Zeocin	IgG	pTRIOZ-mIgG1e2	ExpiFectamine/Microfluidic sorting	32–166	~ 35–37	This study

*CHO* Chinese hamster ovary,* IgG* Immunoglobulin G,* MTX* Methotrexate,* LDC* Limiting dilution cloning,* FL* Fluorescence,* ND* Not determined

## Materials and methods

### Chemicals and consumables

Chinese hamster ovary (ExpiCHO-S) cells, ExpiCHO™ Expression Medium (ECEM), ExpiFectamine™ CHO Transfection Kit, Zeocin™ selection reagent, *NotI* restriction enzyme, and CellTrace™ Far Red (CTFR) were obtained from Thermo Fisher Scientific (USA). The pTRIOZ-mIgG1e2 expression vector was purchased from InvivoGen (USA). Succinimidyl ester fluorophores (iFluor^®^ 488 and iFluor^®^ 594) were obtained from AAT Bioquest (USA). Bovine serum albumin (BSA), Tween-20, OptiPrep™, and Pluronic F-68 were purchased from Sigma-Aldrich (USA). PicoBreak™, Cyto-Surf™ A and B oils, and Cyto-Cartridges™ were obtained from Fluidic Sciences (UK). Detailed information on fluorescent probes and labeling characteristics is provided in the Supplementary Materials (Table S1).

### Cell lines and culture conditions

ExpiCHO-S cells (Chinese hamster ovary cells; *Cricetulus griseus*, ovary-derived epithelial cells; female; RRID: CVCL_5J31) were obtained from Thermo Fisher Scientific in 2022 (Cat. No. A29127). This cell line is a commercially available, suspension-adapted derivative of CHO cells optimized for recombinant protein expression. While formal short tandem repeat (STR) authentication is not standard for CHO-derived cell lines, cell identity was maintained according to supplier specifications. To our knowledge, this cell line has not been reported as misidentified or contaminated. Cell cultures were routinely tested and confirmed to be free of mycoplasma contamination using the e-Myco™ Mycoplasma PCR Detection Kit (ver. 2.0, LiliF Diagnostics) according to the manufacturer’s instructions.

Chinese hamster ovary (ExpiCHO-S) cells were maintained in ExpiCHO™ Expression Medium (ECEM) under standard suspension culture conditions (37 °C, 8% CO₂, shaking at 125 rpm). Cells were passaged every 2–3 days to maintain densities between 0.3 and 6 × 10⁶ cells/mL.

Conditioned medium was prepared by culturing ExpiCHO-S cells in ECEM to mid-log phase (3–4 × 10⁶ cells/mL), followed by centrifugation at 300 × g for 5 min and filtration through a 0.22 μm PES membrane. Filtered conditioned medium was aliquoted and stored at − 20 °C until use.

### Plasmids and transfection

The variable domains of the humanized monoclonal antibody gevokizumab (anti-IL-1β) were cloned into the pTRIOZ-mIgG1e2 expression vector (InvivoGen, USA), which drives coordinated expression of antibody heavy and light chains under independent promoters and includes a Zeocin resistance cassette for selection (Supplementary Figure S7). For transient expression experiments, the plasmid was used in circular form. For stable cell line generation, the plasmid was linearized with *Not*I prior to transfection. ExpiCHO-S cells were transfected using the ExpiFectamine™ CHO Transfection Kit according to the manufacturer’s instructions (Thermo Fisher Scientific, USA). For stable transfection, 0.2 µg of linearized plasmid DNA was used per 1 × 10⁶ cells, and Zeocin selection (50 µg/mL) was applied 48 h post-transfection and maintained for 6 days prior to screening.

### Fluorescent labeling and FRET assay development

Detection reagents, including Protein L and Protein G, were conjugated to iFluor^®^ 488 (donor) and iFluor^®^ 594 (acceptor), respectively, using NHS ester chemistry, following the manufacturer’s standard protocol. Additional detection antibodies were purchased from commercial suppliers as specified in the supplementary section (Table S1). The degree of labeling (DOL)-defined as the average number of fluorophores per protein molecule was determined by UV-VIS spectrophotometry (BioTek Synergy Neo2 Hybrid Multimode Reader, Agilent Technologies, Santa Clara, CA, USA) using dye-specific extinction coefficients and protein absorbance at 280 nm.

To identify optimal donor-acceptor combinations for IgG detection, FRET pair screening was conducted in 384-well microtiter plates using a mouse IgG standard (Sigma) at concentrations ranging from 0 to 166 nM. The donor (488-labeled molecule) was maintained at a constant concentration (166 nM) and mixed with the acceptor (594-labeled molecule) at increasing concentrations to achieve donor-to-acceptor molar ratios of 1:1 (166 nM), 1:2 (332 nM), 1:5 (830 nM), and 1:10 (1660 nM). Donor fluorescence (emission at 520 nm) and FRET signal (emission at 630 nm) were measured using a microplate reader (BioTek Synergy Neo2 Hybrid Multimode Reader, Agilent Technologies, Santa Clara, CA, USA). Signal ratios were calculated to assess FRET efficiency across antibody concentrations and reagent ratios.

### Microfluidic droplet screening and sorting

Droplet-based single-cell encapsulation, incubation, fluorescence interrogation, and sorting were performed using the Cyto-Mine^®^ Single Cell Analysis and Sorting System (Fluidic Sciences, UK). ExpiCHO-S cells were washed three times with PBS (300 × g, 5 min) and resuspended at a density of 0.5 × 10⁶ cells/mL in ExpiCHO™ Expression Medium (ECEM) supplemented with 16% OptiPrep™ and 10% Pluronic F-68. Cells were then mixed with the optimized FRET detection reagent cocktail consisting of 166 nM of both Alexa Fluor^®^ 488-labeled anti-mouse IgG Fc F(ab’)₂ fragment and Alexa Fluor^®^ 594-labeled anti-mouse IgG F(ab’)₂ fragment.

Prior to loading into Cyto-Cartridges™, cell suspensions were filtered through a 35 μm cell strainer to remove aggregates and then encapsulated into aqueous droplets of approximately 95 μm diameter (~ 450 pL), yielding ~ 1.5 × 10⁶ picodroplets per run. Encapsulated cells were incubated on-chip at 37 °C for 60 min to allow antibody secretion and formation of donor-acceptor ternary complexes.

Fluorescence detection was performed using FRET, whereby excitation of the donor fluorophore at 488 nm resulted in energy transfer to the acceptor fluorophore and emission detected at ~ 620 nm (Fig. 2). Fluorescence thresholds were defined based on the peak average intensity distributions of empty and non-secreting picodroplets to distinguish background from true secretion signals. Picodroplets exceeding this threshold were gated on scatter plots and sorted into a dedicated collection chamber. Sorting was performed in two sequential modes: bulk sorting for enrichment of antibody-secreting cells, followed by single-cell sorting for monoclonal clone isolation.

For validation using artificial libraries, transiently transfected ExpiCHO-S cells expressing gevokizumab were stained with CellTrace™ Far Red (CTFR), while non-transfected ExpiCHO-S cells remained unstained. A total of 5 × 10⁵ cells were mixed at defined ratios (5% CTFR-positive and 95% CTFR-negative) prior to encapsulation. The CTFR signal is spectrally distinct from the donor (520 nm) and FRET (620 nm) channels and was therefore used exclusively for post-sort flow cytometry-based quantification of enrichment without interfering with droplet-based FRET detection.

### Droplet recovery and flow cytometry

For bulk-sorted artificial libraries or first round of transfected cell populations, sorted picodroplets were recovered following the manufacturer’s pooled dispensing and cell recovery protocol. Picodroplets were first pooled and dispensed into well A1 of a 24-well microtiter plate containing 1 mL of fresh serum-free culture medium supplemented with 16% (v/v) OptiPrep™. The entire contents of the well were then transferred to a sterile 5 mL tube and allowed to stand upright and undisturbed for 1 min to enable phase separation. The bottom oil phase was carefully removed, and the remaining emulsion phase was demulsified by adding 1 mL of PicoBreak™ reagent (Fluidic Sciences, UK) followed by gentle inversion of the tube several times. After allowing the mixture to settle for 2 min to complete de-emulsification, the lower PicoBreak™ phase was removed. The upper aqueous phase containing the recovered sample was transferred to a 15 mL tube, diluted fivefold with fresh medium, and centrifuged at 300×g for 20 min. Finally, the supernatant was carefully removed without disturbing the cell pellet, and the recovered cells were resuspended in ECEM for downstream analysis. For the first-round bulk-sorted transfected cell population, recovered cells were cultured in ECEM under antibiotic selection ExpiCHO-S culture conditions for 14 days to allow recovery and expansion prior to the second round of single-cell sorting. Cell viability was routinely monitored using an automated trypan blue cell counter during this period. Enrichment of antibody-secreting cells from artificial libraries was evaluated by flow cytometry using an LSRFortessa™ analyzer (BD Biosciences, San Jose, CA, USA) by quantifying CTFR-positive (producer) and CTFR-negative (non-producer) cell populations.

In contrast, when droplets were sorted directly into 96-well plates for single-cell cloning, no demulsification step was performed, as individual droplets were dispensed intact into culture medium to enable direct single-cell outgrowth.

### Single-cell survival condition optimization

To evaluate post-sorting viability and single-cell outgrowth, ExpiCHO-S cells were sorted using a FACSAria™ Fusion flow cytometer (BD Biosciences, San Jose, CA, USA) equipped with a 488 nm laser, as described previously [38]. Forward scatter (FSC) and side scatter (SSC) parameters were used to identify the main cell population, and singlets were gated using FSC-A versus FSC-H to exclude doublets and aggregates. Single, viable cells were dispensed at a density of one cell per well into 96-well plates (five plates per condition) prefilled with 50 µL of one of the following media: ExpiCHO™ Expression Medium (ECEM), ECEM supplemented with 2.5% hypoxanthine-thymidine (HT), or low-cell-density (LCD) medium consisting of 50% conditioned ECEM, 47% fresh ECEM, 2% glutamine, and 1% HT supplement (v/v). After sorting, wells were topped up to a final volume of 150 µL.

Plates were incubated at 37 °C in a humidified atmosphere containing 8% CO₂. Single-cell survival and colony outgrowth were assessed on day 14 using a Scienceware^®^ plate reader magnifying mirror. (Sigma-Aldrich, St. Louis, MO, USA).

### Antibody quantification and productivity assessment

Antibody secretion was quantified by ELISA using purified mouse gevokizumab IgG as a standard. High-binding 96-well plates (Corning™) were coated with recombinant feline IL-1β antigen (1ug/mL), and bound antibodies were detected using a goat anti-mouse IgG-HRP secondary antibody (SouthernBiotech) at a 1:15,000 working dilution, followed by development with TMB substrate. Absorbance was measured at 450 nm using a BioTek Synergy Neo2 plate reader (Agilent Technologies). Culture media (ECEM) was included to determine the assay background, while culture supernatant from non-transfected ExpiCHO-S cells served as the negative control.

Total antibody titers (mg/L) were calculated from standard ELISA curves generated with protein A affinity and SEC purified antibody. Specific productivity (pg/cell/day) was determined based on viable cell counts and culture volumes collected over a 90 h production interval. For scale-up assessment, selected clones were expanded to 25 mL shake flasks and re-evaluated at 7-day intervals. Clone stability was monitored over 30 days of continuous culture without selection pressure.

### Data analysis

Flow cytometry data and microfluidic data were analyzed and plotted using FlowJo software (version 10.10, BD Biosciences, San Jose, CA, USA). ELISA data analyses were performed using GraphPad Prism (version 10.4.1, GraphPad Software, San Diego, CA, USA). Figure schematics were created using BioRender.com.

The authors used ChatGPT (OpenAI, San Francisco, CA, USA) for grammar and language correction assistance during manuscript preparation. The tool was not used for generating scientific content, data analysis, or interpretation.

Murine IgG was used here as a representative model antibody to establish and validate the workflow; however, the same assay development principles and droplet screening strategy are readily transferable to other antibody formats by selecting appropriate species- or isotype-specific detection reagents. More broadly, the workflow should be adaptable to additional secreted recombinant proteins, provided that compatible secretion-detection chemistries can be established. This broader applicability is a key motivation for detailing the assay-development process in the present study. In this context, the assay-development strategy presented here is intended to serve as a practical guide for adapting FRET-based secretion assays to other recombinant proteins of interest, including selection of appropriate binding reagents, optimization of donor-to-acceptor ratios, definition of the relevant concentration window, and evaluation of signal saturation or hook-like behavior.

In conclusion, this study establishes a practical and reproducible workflow for rapid stable CHO cell line development secreting high levels of antibodies by integrating secretion-based screening, image-verified monoclonality, and early clonal recovery within a droplet microfluidic format. By reducing reliance on prolonged iterative screening and enabling earlier identification of productive monoclonal cells, this approach provides a streamlined strategy for accelerating upstream CLD. While subsequent strain and process optimization remain necessary for manufacturing-scale production, the workflow described here offers a robust and adaptable foundation for efficient early-stage mammalian CLD.

## Supplementary Information

Below is the link to the electronic supplementary material.

Supplementary Material 1

## Data Availability

The datasets used and/or analyzed during the current study are available from the corresponding author on reasonable request.
