# Supplementary material for: Streamlined stable CHO cell line development using droplet microfluidics with image-based monoclonality assessment
Source: Bioprocess Biosyst Eng. 2026 Aug 19;49(10):2703–19. doi: 10.1007/s00449-026-03406-7 (PMC13582368; doi:10.1007/s00449-026-03406-7)
Supplement: Supplementary file 1 [file 449_2026_3406_MOESM1_ESM.docx]

**Supplementary Materials**

**Streamlined stable CHO cell line development using droplet microfluidics with image-based monoclonality assessment**

Kumar Vishven Naveen^1^, Akanksha Tyagi^1^, Omnia Mohammed Hamid Ibrahium^2^, Rainer EAW Fischer^1^, and Raluca Ostafe^1,2*^

^1^Purdue Institute of Inflammation, Immunology and Infectious Disease, Purdue University, West Lafayette, 47907, United States

^2^Department of Biological Sciences, Purdue University, West Lafayette, IN 47907, United States

* Corresponding author- rostafe@purdue.edu

**Table S1**. List of donor/acceptor probe pairs evaluated for FRET-based total IgG detection, including full-length antibodies (anti-Fc and anti-F(ab')₂), F(ab')₂ fragments, and Protein G- or Protein L-based binding reagents.

| **Ref.** | | **Species** | **Antigen** | **Fluorophore** | **Ab Type** | **Degree of Labeling (DOL)** | **Supplier** | **Product #** |
| --- | --- | --- | --- | --- | --- | --- | --- | --- |
| Figure 3a | Donor | Goat | Mouse IgG, F(ab')_2_ | Alexa Fluor® 488 | IgG, Polyclonal | 1.648 | Jackson Immuno Research | 115-545-006  lot 174859 |
|  | Acceptor | Goat | Mouse IgG, Fcγ | Alexa Fluor® 594 | IgG, Polyclonal | 1.228 | Jackson Immuno Research | 115-585-008  lot 174840 |
| Figure 3b | Donor | Goat | Mouse IgG, F(ab')_2_ | DyLight® 488 | F(ab')_2_, Polyclonal | 1.305 | Abcam | ab98757 |
|  | Acceptor | Goat | Mouse IgG, Fc | DyLight® 594 | IgG, Polyclonal | 3.055 | Abcam | ab97017 |
| Figure 3c | Donor | *E. coli* | IgG, Fc | iFluor® 488 succinimidyl ester (staining in house) | Protein G | 2.42 | GenScript | Z02007 |
|  | Acceptor | *E. coli* | IgG, κ (kappa) light chains | iFluor® 594 succinimidyl ester (staining in house) | Protein L | 0.987 | Pierce | 21189 |
| Figure 3d | Donor | Goat | Mouse IgG, Fcγ | Alexa Fluor® 488 | F(ab')_2_, Polyclonal | 1.676 | Jackson Immuno Research | 115-546-008  lot 169468 |
|  | Acceptor | Goat | Mouse IgG, F(ab')_2_ | Alexa Fluor® 594 | F(ab')_2_, Polyclonal | 0.642 | Jackson Immuno Research | 115-586-006  lot 169591 |

**Figure S1**. Flow cytometry analysis of artificial mixed-library CHO-S cell populations used to evaluate enrichment by picodroplet sorting. (a) CellTrace™ Far Red (CTFR)-stained CHO-S cells transiently expressing gevokizumab, used to define the producer population prior to library preparation. (b) Unstained non-expressing CHO-S cells used to define the non-producer population. (c) Artificial mixed library generated by combining CTFR-positive and unstained cells at a 5%:95% ratio prior to picodroplet sorting. (d) Flow cytometry analysis of the enriched cell population immediately after picodroplet sorting, demonstrating enrichment of CTFR-positive antibody-secreting cells.

**Figure S2.** Single-cell outgrowth of ExpiCHO-S cells following deposition into 96-well plates using a FACSAria™ Fusion sorter. Cells were deposited into 200 µL/well of the indicated media and outgrowth was assessed on Day 14. For each medium condition, cells were deposited into five 96-well plates, corresponding to up to 480 single-cell wells per condition. Outgrowth was calculated as the percentage of wells showing viable colony recovery. Error bars represent the standard deviation across five plates (n = 5 plates). ECEM, ExpiCHO Expression Medium; HT, HT supplement; LCD, Low Cell Density medium.

**Figure S3**. Kill-curve analysis of ExpiCHO-S cells treated with Zeocin (0–500 µg/mL), with cell viability measured at specified time points. Viability was determined using Trypan blue. C; control

**Figure S4.** Representative images of picodroplets prior to dispensing. Five sequential images of each picodroplet were recorded immediately before dispensing into a well of a microtiter plate. Shown here is picodroplet from sorting event 1, sample 18 on plate 3 (well 3B7), containing a single cell. The accompanying scatterplot indicates the location of this picodroplet.

**Figure S5.** ELISA-based screening of antibody production in individual clones following single-cell sorting and 7 days of recovery period, under Zeocin selection. The x-axis numbers represent individual single-cell-derived cultures/wells in the order in which they were screened by ELISA. The ELISA positivity threshold was defined for the assay as the mean signal of the non-transfected negative control plus three standard deviations (mean + 3 SD). Clones with signals above this threshold were considered antibody-producing. Positive clones were subsequently ranked according to their ELISA signal, and clones with the highest signals were selected for further characterization. This ELISA-based distribution also reflects clone-to-clone variability in antibody accumulation after single-cell recovery.

**Figure S6.** Characterization of representative monoclonal ExpiCHO-S antibody-producing CHO clones. (a) Specific productivity (qP) of three representative monoclonal ExpiCHO-S clones (Clone 1, Clone 2, and Clone 3) following clonal expansion. (b) Corresponding volumetric antibody production of the same clones, determined by ELISA.

**Figure S7**. Map of Gevokizumab expression vector pTRIOZ-mIgG1e2.
